# The Antioxidant Potential of White Wines Relies on the Chemistry of Sulfur-Containing Compounds: An Optimized DPPH Assay

**DOI:** 10.3390/molecules24071353

**Published:** 2019-04-05

**Authors:** Remy Romanet, Christian Coelho, Youzhong Liu, Florian Bahut, Jordi Ballester, Maria Nikolantonaki, Régis D. Gougeon

**Affiliations:** 1 Univ. Bourgogne Franche-Comté, AgroSup Dijon, PAM UMR A 02.102, Institut Universitaire de la Vigne et du Vin, Jules Guyot, Rue Claude Ladrey, BP 27877, 21078 Dijon CEDEX, France; remy.romanet@u-bourgogne.fr (R.R.); christian.coelho@u-bourgogne.fr (C.C.); Florian.Bahut@u-bourgogne.fr (F.B.); maria.nikolantonaki@u-bourgogne.fr (M.N.); 2 Current address: Department of Mathematics and Computer Science, Advanced Database Research and Modelling (ADReM), University of Antwerp, 2020 Antwerp, Belgium; Youzhong.Liu@uantwerpen.be; 3 Centre des Sciences du Goût et de l’Alimentation, AgroSup Dijon, CNRS, INRA, Université de Bourgogne-Franche-Comté, 9 E Boulevard Jeanne d’Arc, F-21000 Dijon, France; jordi.ballester@u-bourgogne.fr

**Keywords:** DPPH, antioxidant capacity, Chardonnay, white wine, EC_20_, sensory oxidation level, sulfur compounds

## Abstract

The DPPH (2,2-Diphenyl-1-picrylhydrazyl) assay is an easy and efficient method commonly used to determine the antioxidant capacity of many food matrices and beverages. In contrast with red wines, white wines are poorer in antioxidant polyphenolics, and the more hydrophilic sulfur-containing compounds in them may contribute significantly to their antioxidant capacity. The modification of the classical DPPH method, with a methanol-buffer and the measure of EC_20_ (quantity of sample needed to decrease the initial DPPH concentration by 20%) has shown that sulfur-containing compounds such as cysteine (0.037 ± 0.003), glutathione (0.054 ± 0.003) or methanethiol (0.104 ± 0.003) appeared to bear antioxidant capacity comparable to well known phenolic compounds, such as catechin (0.035 ± 0.003), caffeic acid (0.057 ± 0.003) and ferulic acid (0.108 ± 0.003), respectively. In the case of white wines, the comparison with REDOX-sensory scores showed that results from this modified DPPH assay are strongly correlated with sensory attributes (r = 0.73, *p* < 0.1). These results provide an unprecedented illustration of the important contribution of these sulfur-containing compounds to the radical quenching ability of white wines.

## 1. Introduction

The aging of premium wines, and in particular dry white wines, has become an important scientific issue over the last decades, where the phenomenon called “premature aging” has appeared worldwide. In the context of sustainability, new processes based on the reduction of inputs, both at the vineyard and in the cellar (for instance sulfites), are sometimes considered as threats towards expected organoleptic optimums. Therefore, a better understanding of the physico-chemical mechanisms of oxidation is required to predict and control the appearance of premature aging. This is based in particular on the development of innovative predictive tools for determining the oxidative stability of a wine.

Wine oxidative stability can be related to intrinsic and extrinsic factors that prevent aroma deterioration [1]. Up to now, studies dealing with white wine antioxidant capacity were mostly focused on the description of the antioxidant effects of sulfites or glutathione via monitoring the evolution of potent volatile oxidation markers, but without giving any insights into the mechanisms controlling their formation [1]. In such a complex matrix as white wine, the role of each naturally present antioxidant is hard to establish, and as a consequence, the sole targeted analysis of known key antioxidant compounds (polyphenols and sulfur compounds) is poor at predicting the evolution of the wine. In that respect, we have shown clearly that the antioxidant metabolome of Chardonnay white wines is highly dependent on the management of N–S-containing compounds from the very beginning of the winemaking process, through the comparison of glutathione additions at early and late stages [2]. These results highlight the need for a method that can assess the instantaneous antioxidant potential of the global wine metabolome. Up to now, voltammetry and EPR (electron paramagnetic resonance) spectroscopy based methodologies have been proposed to measure wine antioxidant potential [3,4]. The latter gives a fine classification of wines according to their antioxidant potential, but is expensive and demands an expertise in data acquisition and treatment, whereas voltammetry mostly targets polyphenols.

The DPPH (2,2-Diphenyl-1-picrylhydrazyl) assay is a simple, inexpensive and efficient method, one of the most commonly used to determine the antioxidant capacity of a compound, an extract or other biological matrices (polyphenols, plants, fruits, wine…) [5,6,7,8]. DPPH is a stable free radical that is reduced by antioxidant molecules, by acceptation of an electron or a hydrogen [7,9]. In the initial radical form, DPPH has a deep purple color, which changes to yellow in the reduced form. DPPH has an important absorption band in the range 515–520 nm, which makes spectrophotometry an easy tool to measure the color change and determine the antioxidant activity of the sample. The more this color changes, the more DPPH is reduced and the better the antioxidant activity of the sample is. The antioxidant capacity is commonly expressed as the efficient concentration (EC_50_), which represents the quantity of the sample needed to decrease the initial concentration of the DPPH free radical by 50%. However, some studies showed the lack of correlation between the DPPH discoloration and the concentration of the substrate [9,10,11,12]. In order to achieve a better linearity, a statistical software was developed by Locatelli et al. (2009) by using different regression models. In order to stay in the linear range, determining the EC_20_, representing the quantity of antioxidant needed to decrease the initial concentration of DPPH by 20%, was proposed [5].

It has been shown that DPPH can be reduced by amino acids containing cysteine and aromatic amines [13]. Because of the hydrophobic character of DPPH, the DPPH assay is generally adapted to methanol solvent [5,6,7,9]. In contrast with white wines, which bear low concentrations of polyphenols, the antioxidant capacity of polyphenol rich red wines matches the strong lipophilic character of DPPH well [6]. This implies that other compounds, which may play a more important role in the antioxidant capacity of white wines—because of higher concentrations relative to the phenolic concentration—are not taken into account by the conventional methanol measurement of the DPPH assay. It must be noted that some researchers have already used methanol-buffer mixtures to prepare DPPH solution to solve this problem, but using acetate buffer at a pH between 4.5 and 5.5, which is higher than the pH of wine [8,14,15].

In order to estimate the shelf life aging potential of white wines, on the basis of a more genuine antioxidant potential, the present paper reports the development of a predictive tool based on a revisited DPPH assay adapted to white wine matrices. For optimization of the DPPH assay, the antioxidant capacity of selected active compounds present in white wines, especially N–S-containing compounds and polyphenols, was compared in buffers with varying polarities.

## 2. Results and Discussion

### 2.1. Buffer Optimization

**Figure 1:**
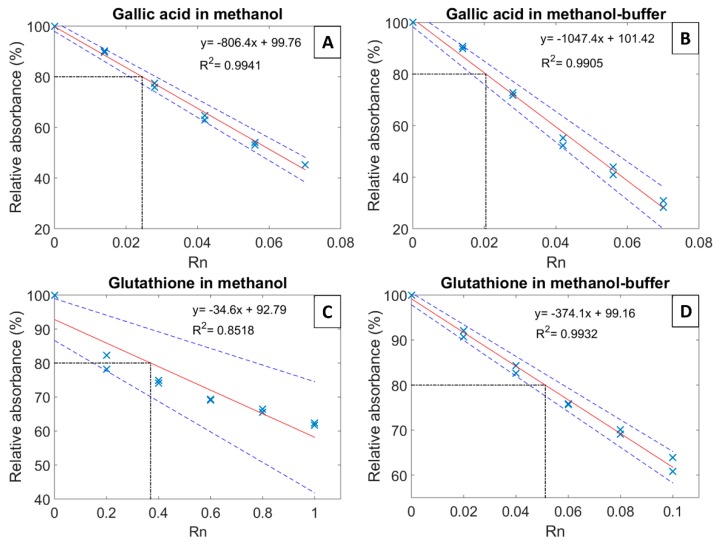
DPPH measurements of gallic acid (**A**,**B**) and glutathione (**C**,**D**) in methanol and in methanol-buffer (0.1 M of citric acid and 0.2 M of phosphate disodium, pH 3.6, final proportion 60% methanol and 40% buffer). The plain line (red) represents the regression line, and the two dashed lines (blue) the 95% confidence interval. The dot-dashed line guides the eye to EC_20_, which is the Rn value corresponding to a 20% decrease of the initial absorbance. Rn represents the ratio between the molar number of standard compound (in mol) added to the initial molar number of DPPH (in mol).

The solvent optimization for the DPPH assay adapted to white wines was carried out by comparing gallic acid and glutathione as active compounds. To that purpose, methanol and methanol-buffer (0.1 M of citric acid and 0.2 M of phosphate disodium, pH adjusted to 3.6, final proportion 60% of methanol and 40% buffer) were tested as reaction solvents (Figure S1 shows the UV-Vis absorbance of DPPH). Figure 1 shows the antioxidant capacity (EC_20_) of both gallic acid and glutathione measured in the two tested solvents. EC_20_ is the Rn value corresponding to a 20% decrease of the initial absorbance, where Rn represents the ratio between the molar number of standard compound (n_compound_ mol in 100 μL) added to the initial molar number of DPPH (n_DPPH_ mol in 3.9 mL). Gallic acid gave similar and reproducible results with the two solvents, with an EC_20_ of 0.025 ± 0.004 and 0.020 ± 0.005 for methanol and methanol-buffer, respectively (Figure 1a,b). In contrast, when measured in methanol, the EC_20_ of glutathione appeared to be of the order of 0.4 with a rather large uncertainty (non-linearity), compared to 0.051 ± 0.006 and with good linearity in methanol-buffer solvent (Figure 1c,d). The difference between glutathione solubility in methanol (1.20 ± 0.03 g/L) and in methanol-buffer (12.30 ± 0.06 g/L) could certainly explain the discrepancy between the two latter measurements and the lack of linearity when methanol was used as reaction solvent (Figure 1c).

**Figure 2:**
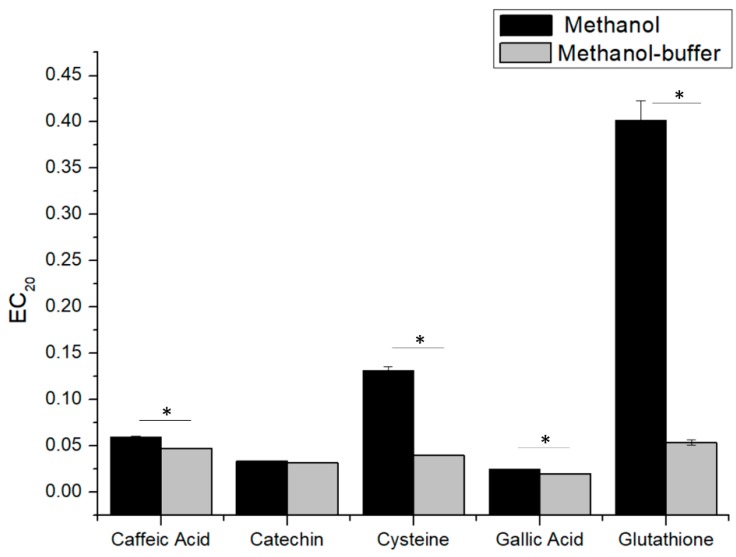
EC_20_ values of gallic acid, catechin, caffeic acid, glutathione and cysteine measured in methanol (■) and in methanol-buffer (■). Student tests show comparison between methanol and methanol-buffer methods, “*” indicates significant differences (*p* < 0.05).

In order to confirm methanol-buffer acceptance for estimating white wine antioxidant capacity, EC_20_ values were measured for a large variety of key antioxidant compounds. Figure 2 shows the comparison of EC_20_ values for gallic acid, catechin, caffeic acid, glutathione and cysteine. As observed previously for gallic acid, all tested phenolic compounds presented good reproducibility and consistency in both methanol and methanol-buffer conditions (except for caffeic acid), while, for sulfur containing compounds (glutathione and cysteine), there was a clear discrepancy between the two solvents, and the EC_20_ results had good repeatability (CV% lower to 5%) only using methanol-buffer. These results clearly highlight the correlation between the solubility and the genuine DPPH measurement, where the actual EC_20_ determined for glutathione and cysteine was much smaller (higher related antioxidant capacity) than the one measured in methanol, due to a better solubility of the compounds in the buffer. In agreement with these results, methanol-buffer was chosen to determine the antioxidant capacity of wine-relevant antioxidants and white wines by our modified DPPH assay.

### 2.2. Classification of White Wine Relevant Antioxidants Based on Our Modified DPPH Assay

Phenolic compounds and thiols are the two most important antioxidants in white wines. The antioxidant power of polyphenols is assigned to their prompt reaction with reactive oxygen species (ROS) at pH of wine, while their generated quinonic forms are highly electrophilic and responsible for the generation of oxidative aroma notes [16,17]. On the other hand, thiols are preservatives by virtue of their ability to act as quinone reductants and/or scavengers and are considered key factors that govern wine resistance to oxidative aging [2,18,19].

**Figure 3:**
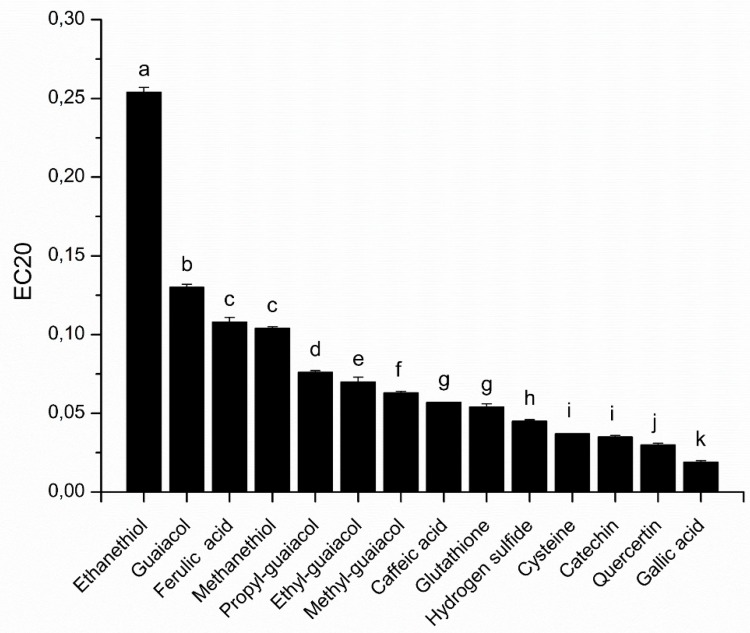
Comparison of the antioxidant capacity (EC20) of selected wine compounds using DPPH in methanol-buffer. Results of ANOVA and Tukey HSD tests: Different letters mark significant differences at *p* < 0.05.

In our study, phenolic compounds from different families (hydroxycinnamic acids, benzoic acids, flavanols and flavonols) and thiols (amino acids, volatile sulfur compounds) were assessed by the proposed DPPH assay to estimate their antioxidant effect in wine like conditions (Figure 3).

Under our experimental conditions, among the tested phenolic compounds, gallic acid presented the highest antioxidant capacity (EC_20_ = 0.019 ± 0.001), followed by quercetin and catechin, presenting EC_20_ values of 0.030 ± 0.001 and 0.035 ± 0.001, respectively. This classification is in accordance with the literature when the solvent used was ethanol or methanol. However, the value of EC_20_, or the equivalent EC_50,_ are not similar due to differences in the concentration of DPPH used [5,11,12]. Among the tested thiols, cysteine, H_2_S and glutathione exhibited the higher antioxidant capacity, with an EC_20_ of 0.037 ± 0.000, 0.045 ± 0.001 and 0.054 ± 0.002, respectively. Cysteine showed no significant difference in antioxidant capacity compared with catechin. Moreover, glutathione and caffeic acid (EC_20_ = 0.057 ± 0.000) also exhibited similar antioxidant capacity. These results show the importance of sulfur compounds in the trapping of free radicals, and therefore as contributors to the antioxidant capacity of wines. Glutathione which is a tripeptide composed of glutamic acid, cysteine and glycine has an important antioxidant capacity, which shows that it plays an important role in antioxidant mechanisms involved in wines. However, it is noteworthy that cysteine, which is the reactive amino acid of glutathione, displays a better antioxidant capacity, which would be due to steric hindrance of glutathione against DPPH.

The comparison of the antioxidant capacity of volatile sulfurs, such as hydrogen sulfide (H_2_S), methanethiol and ethanethiol, which have an EC_20_ of 0.045 ± 0.001, 0.104 ± 0.001 and 0.254 ± 0.003, respectively, shows that their antioxidant capacity could be correlated to the pKa of the corresponding thiol (pKa = 7.04 for H_2_S, 10.4 for methanethiol and 10.6 for ethanethiol). When pKa increases, the liberation of a hydrogen is more difficult and the quenching of the DPPH radical is more difficult. Interestingly, our modified DPPH assay revealed that volatile thiols, such as methanethiol, could exhibit comparable antioxidant capacity (EC_20_) to a phenolic, such as ferulic acid.

Moreover, our results also showed that the addition of an alkyl group to the guaiacol moiety increases the antioxidant capacity of the compounds. Indeed, the EC_20_, which is 0.130 ± 0.002 for guaiacol, decreases to 0.076 ± 0.001, 0.070 ± 0.003 and 0.063 ± 0.001 for propyl, ethyl and methyl-guaiacol, respectively, which would be due to an increase in the number of limit forms for the reactive intermediate.

### 2.3. Antioxidant Capacity of White Wines

**Figure 4:**
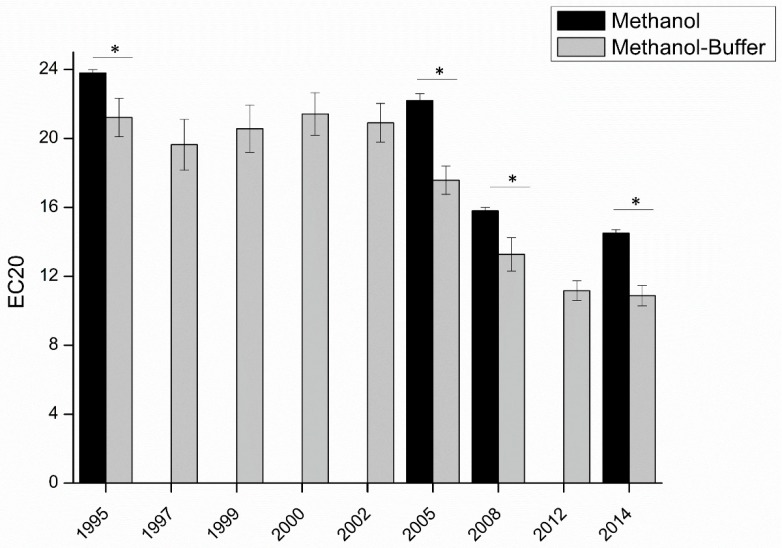
Comparison of the antioxidant capacity of the same appellation from nine vintages from the same winery. Measurements in methanol (■), and in methanol-buffer (■). Student tests show comparison between methanol and methanol-buffer methods, “*” indicate significant difference (*p* < 0.05).

In order to apply this modified DPPH methodology to the measurement of the antioxidant capacity of white wines, to understand their oxidative stability, nine vintages for a given wine from the same plot and the same winery were analyzed, four of which were also analyzed using the classical methanol solvent for comparison (Figure S2 and Figure 4). In agreement with the expected hierarchy in terms of antioxidant concentrations, younger wines presented a better antioxidant capacity than older ones.

Secondly, the EC_20_ obtained with the classical methanol method was always significantly higher than that with the methanol-buffer method. In agreement with measures on standard compounds (Figure 2), this result can be explained by the fact that with methanol, the measured antioxidant capacity of the wine is mostly driven by the reactivity of phenolic compounds. Instead, using the methanol-buffer method, the contribution of other compounds, and in particular thiols, like glutathione, would be allowed. On that basis, the highest difference between EC_20_ determined using methanol and methanol-buffer for the 2005 vintage (difference of 4.7, compared to 2.5 for 1995, 2.5 for 2008, and 3.6 for 2014) could be evidence of the relatively higher concentrations of sulfur compounds in this vintage.

### 2.4. Comparison to Sensory Oxidation Levels

REDOX sensory scores, as well as intensity scores of the most cited attributes (at least by 15% of the panel for at least one sample), were submitted to ANOVA with the vintage as a fixed factor, the panelist as a random factor and the interaction vintage*panelist used as an error term. The results are presented in Table 1, with REDOX sensory score, oaky, caramel/vanilla, roasted, vegetables, dust/carboard bruised apple and cork showing significant vintage effects. Oaky, caramel/vanilla, roasted also showed significant interactions, indicating some disagreement among panelists regarding these attributes.

In order to explore possible correlations between our measured antioxidant capacity (EC_20_) and sensory properties of the seven wines, a principal component analysis (PCA) was carried out including EC_20_ mean values and the sensory attributes with a vintage *p*-value lower than 0.1.

**Figure 5:**
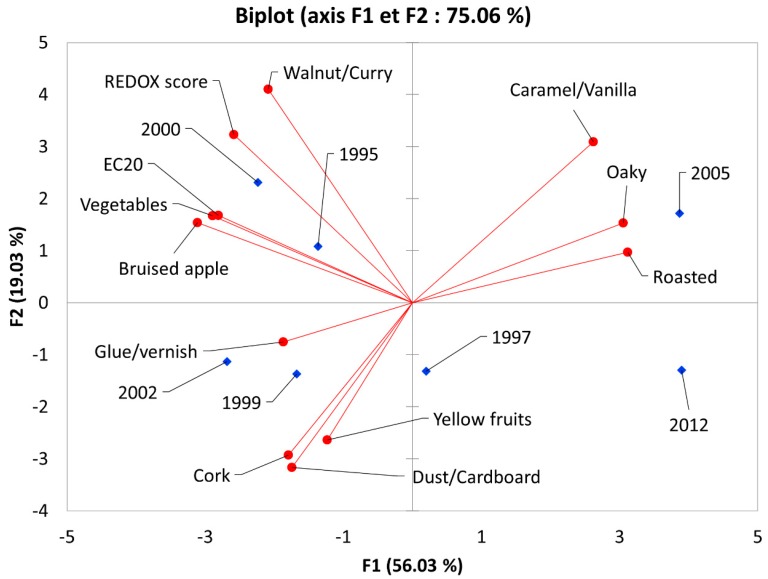
Principal component analysis (PCA) biplot representing the oxidation levels determined by sensory analyses and revisited DPPH measurements.

The biplot (Figure 5) shows a clear segmentation between the youngest wines (2005 and 2012) characterized by oak ageing notes and the other wines at the opposite side of F1 characterized either by oxidation (2000, 1995) or fruity notes and some presence of cork taint (2002, 1999). The sample from 1997 stands in the middle of the F1–F2 plot and is more associated with yellow fruits in F3 (data not given). These results show that, in agreement with previous literature, the oxidative status of a wine is not always correlated to its age since strong vintage effects can occur [20,21]. Moreover, EC_20_ showed strong Pearson correlations with oxidative attributes like bruised apple (r = 0.84, *p* < 0.05), walnut/curry (r = 0.82, *p* < 0.05), and the REDOX sensory score (r = 0.73, *p* < 0.1).

## 3. Materials and Methods

### 3.1. Chemicals

1,1-Diphenyl-2-picrylhydrazyl radical (DPPH), citric acid, sodium phosphate dibasic, quercetin, catechin, caffeic acid, guaiacol, methyl-guaiacol, ethyl-guaiacol, propyl-guaiacol, ferulic acid, sodium sulfide, sodium thiomethoxide, ethanethiol, and glutathione were purchased from Sigma-Aldrich (St. Louis, MO, USA). Gallic acid and cysteine were purchased from Merck. Methanol was purchased from Chemlab. Ethanol was purchased from Honeywell. Ultrapure water was obtained from a Milli-Q system.

### 3.2. Antioxidant Capacity of Standard Compounds

The phenolic compounds were dissolved in water/ethanol (50/50) solutions. The other compounds were dissolved in water. The solutions were then diluted with water to obtain different concentrations. Ethanol and water were first oxygen degassed with Argon. All dilutions were prepared in a glove box to be in a controlled atmosphere without oxygen. All the solutions were prepared freshly before analysis.

### 3.3. Wines

Nine chardonnay wines from the Burgundy region and for different vintages (between 3 and 22 years old) were selected for DPPH assays. Before analysis, and since sulfites can react with DPPH and contribute to the antioxidant capacity of white wines [14,22], these nine chardonnay wines were degassed using CO_2_ (3mL/min) to remove free SO_2_ as suggested by Pegram et al. (2013) [23].

### 3.4. Methods

The optimum DPPH solution was prepared at 25 mg/L into a mixture of methanol and citrate-phosphate buffer. The citrate-phosphate buffer was prepared with 0.1 M citric acid and 0.2 M phosphate disodium, and the pH was adjusted to 3.6 in order to be close to a wine pH. Methanol was then added to obtain a final mixture containing 60% methanol and 40% buffer. This optimum methanol-buffer concentration was adapted from the literature, where either 60–40% [8,15] or 50–50% [14] ratios were reported, for methanol-acetate buffer. We observed that the ratio 60–40% was the most adapted due to better DPPH stability over time. Samples (100 µL) (standard compounds or wines) were added to 3.9 mL of DPPH solution [6,7]. This preparation was performed in a glove box to be in a controlled atmosphere without oxygen. The reaction time was fixed to 240 min in order to reach a plateau value [5], and the absorbance of the solution was read at 525 nm, considered to be at the maximum of absorption. A molar ratio R_n_ was defined as the ratio between the molar number of standard compound introduced (n_Compound_ in 100 µL) and the molar number of DPPH initially present (n_DPPH_ in 3.9 mL), as:

(1) $$R_{n}=\frac{n_{Compound}}{n_{DPPH}}$$

The normalized absorbance (Abs_%_) was obtained by the difference between the absorbance of the blank (Abs_blank_) and the absorbance of the sample (Abs_sample_) divided by the absorbance of the blank:

(2) $$Abs_{\%}=\frac{Abs_{blank}-Abs_{sample}}{Abs_{blank}}*100$$

The normalized absorbance was then plotted against the molar ratio to determine the Ec_20_ [5], which is the Rn value needed to decrease by 20% the initial absorbance of DPPH (Figure 1).

For the determination of the antioxidant capacity of wines, the molar number (or the concentration) of antioxidant compounds was unknown, therefore the molar ratio R_n_ could not be calculated. Instead, and in order to account for all the antioxidant contributors, the volume V of wine, needed to reduce the initial absorbance by 20% added to DPPH was used. In order to account for the experimental concentration of the DPPH solution ([DPPH]_exp_), the added volume was normalized (*V_norm_*) for a solution at 25 mg/L of DPPH ([DPPH]_th_), as follows:

(3) $$V_{norm}=V*\frac{\;{\left[DPPH\right]}_{th}}{{\left[DPPH\right]}_{exp}}$$

The absorbance was then normalized by the absorbance of a blank (water) and plotted against the corrected volume (*V_norm_*) to determine the EC_20_ of the wines.

### 3.5. Sensory Analysis

Seven out of the nine wines were submitted to sensory analysis. Sixteen oenology students from the University of Burgundy were trained for 2 months specifically on the wine oxidation and reduction aromas. The panel included 4 females and 12 males with an average age of 24.2 ± 1.2 years old. The sessions took place in a sensory room with individual booths. Samples (30 mL) were presented at room temperature in standardized black glasses coded with 3-digits following a Latin-square arrangement. For each sample, the panelists were first asked to rate the intensity of oxidation–reduction (REDOX-sensory score) orthonasally (i.e., by smell only) using the protocol proposed by Ballester et al. (2018) [20]. The scale was structured from −5 (strong reduction), +5 (strong oxidation) and zero (neither reduced nor oxidized) in the midpoint. Afterwards they were asked to describe the odor of the sample by choosing a maximum of five attributes from a list of 19 descriptors (see Table 1) and score their intensity in a 4-point discrete scale.

### 3.6. Statistical Analysis

All measurements were performed in duplicate, and the results were expressed as mean ± standard deviation. Comparisons between methods (methanol and methanol-buffer) were carried out using the Student test, and the classification of antioxidant capacity of standard compounds was studied by ANOVA, followed by Tukey (HSD) test. The sensory results were analyzed using an ANOVA and PCA (principal component analysis). These statistical analyses were done with the software XLSTAT. Figures were plotted using Matlab R2015a (MathWorks) and OriginPro 8 softwares (Originlab Corporation, Wellesley Hills, MA, USA).

## 4. Conclusions

The modification of the classical DPPH method, with a methanol-buffer (0.1 M of citric acid and 0.2 M of phosphate disodium, pH adjusted to 3.6, final proportion 60% methanol and 40% buffer) provided new reference values for the antioxidant capacity of active compounds representative for white wines. In particular, and as a result of a significantly higher solubility in this methanol-buffer, sulfur-containing compounds such as cysteine, glutathione or methanethiol appeared to bear antioxidant capacity comparable to well known phenolic compounds, such as catechin, caffeic acid and ferulic acid, respectively. These results provide an unprecedented illustration of the important contribution of these sulfur-containing compounds to the radical quenching ability of white wines.

The analysis of white wines from different vintages, and at different levels of oxidation, has further permitted validation of the method. Moreover, the comparison with REDOX-sensory scores, showed that results from this modified DPPH assay are strongly correlated with sensory attributes of the same wines. Therefore, such a modified DPPH method not only complies with requirements for the measurement of genuine antioxidant capacity of white wines, but also offers an interesting alternative for developing predictive tools of the ageing ability of white wines.

## Figures and Tables

**Table 1:** Sensory attributes and ANOVA results. Significant P-values (5% level) are highlighted in bold letters.

ATTRIBUTE	VINTAGE		TASTER		VINTAGE * TASTER	
	F	*p*-value	F	*p*-value	F	*p*-value
**REDOX SCORE**	**5.4**	**<0.0001**	**2.2**	**0.010**	0.9	0.721
**CITRUS**	1.1	0.388	**3.0**	**0.001**	0.9	0.618
**BITTER ALMOND**	0.8	0.603	**2.7**	**0.002**	1.0	0.516
**BUTTER**	1.5	0.183	**2.7**	**0.002**	1.0	0.486
**RANCID BUTTER**	0.8	0.584	**2.6**	**0.002**	1.3	0.056
**OAKY**	**14.6**	**<0.0001**	1.5	0.113	**2.3**	**<0.0001**
**CARAMEL/VANILLA**	**3.3**	**0.006**	1.7	0.073	**1.9**	**0.0001**
**WAXY/MOTHBALL**	1.3	0.256	1.7	0.073	**1.4**	**0.019**
**GLUE/VERNISH**	**4.4**	**0.001**	**2.3**	**0.008**	1.2	0.115
**STAGNANT WATER**	1.6	0.146	**2.5**	**0.004**	1.2	0.102
**ROASTED**	**11.8**	**<0.0001**	**2.2**	**0.013**	**4.0**	**<0.0001**
**FLORAL**	1.9	0.096	**2.3**	**0.008**	1.0	0.550
**WHITE FRUITS**	1.2	0.338	**3.8**	**<0.0001**	1.4	0.040
**YELLOW FRUITS**	1.9	0.082	**7.8**	**<0.0001**	0.6	0.992
**VEGETABLES**	**3.5**	**0.004**	1.6	0.079	0.9	0.673
**RANCID HONEY**	1.3	0.251	0.9	0.581	1.0	0.422
**WALLNUT/CURRY**	2.1	0.058	1.2	0.259	1.0	0.448
**DUST/CARDBOARD**	**3.8**	**0.002**	1.0	0.501	1.0	0.574
**BRUISED APPLE**	**2.9**	**0.013**	**5.0**	**<0.0001**	1.2	0.162
**CORK**	**14.6**	**<0.0001**	0.9	0.582	0.4	1.000

## Supplementary Materials

The following are available online. Figure S1 UV-Vis absorbance spectra of DPPH in methanol and methanol-buffer, Figure S2 DPPH measurements for two vintages (1999 and 2012) in methanol-buffer.
