# Supplementary material for: The Antioxidant Potential of White Wines Relies on the Chemistry of Sulfur-Containing Compounds: An Optimized DPPH Assay
Source: Molecules. 2019 Apr 5;24(7):1353. doi: 10.3390/molecules24071353 (PMC6479956; doi:10.3390/molecules24071353)
Supplement: Supplementary file 1 [file molecules-24-01353-s001.pdf]

Supplementary Materials

The Antioxidant Potential of White Wines Relies on the Chemistry of Sulfur-Containing Compounds: an Optimized DPPH Assay

Remy Romanet [1], Christian Coelho [1], Youzhong Liu [2], Florian Bahut [1], Jordi Ballester [3], Maria Nikolantonaki [1] and Régis D. Gougeon [1,*]

[1] Univ. Bourgogne Franche-Comté, AgroSup Dijon, PAM UMR A 02.102, Institut Universitaire de la Vigne et du Vin, Jules Guyot, Rue Claude Ladrey, BP 27877, 21078 Dijon Cedex, France; remy.romanet@u-bourgogne.fr (R.R.); christian.coelho@u-bourgogne.fr (C.C.); Florian.Bahut@u-bourgogne.fr (F.B.); maria.nikolantonaki@u-bourgogne.fr (M.N.)

[2] Current address: Department of Mathematics and Computer Science, Advanced Database Research and Modelling (ADReM), University of Antwerp, 2020 Antwerp, Belgium; Youzhong.Liu@uantwerpen.be

[3] Centre des Sciences du Goût et de l'Alimentation, AgroSup Dijon, CNRS, INRA, Université de Bourgogne-Franche-Comté, 9 E Boulevard Jeanne d'Arc, F-21000 Dijon, France; jordi.ballester@u-bourgogne.fr

* Correspondence: regis.gougeon@u-bourgogne.fr

Keywords: DPPH; Antioxidant capacity; White wine; EC_{20}; Sensory oxidation level; sulfur compounds

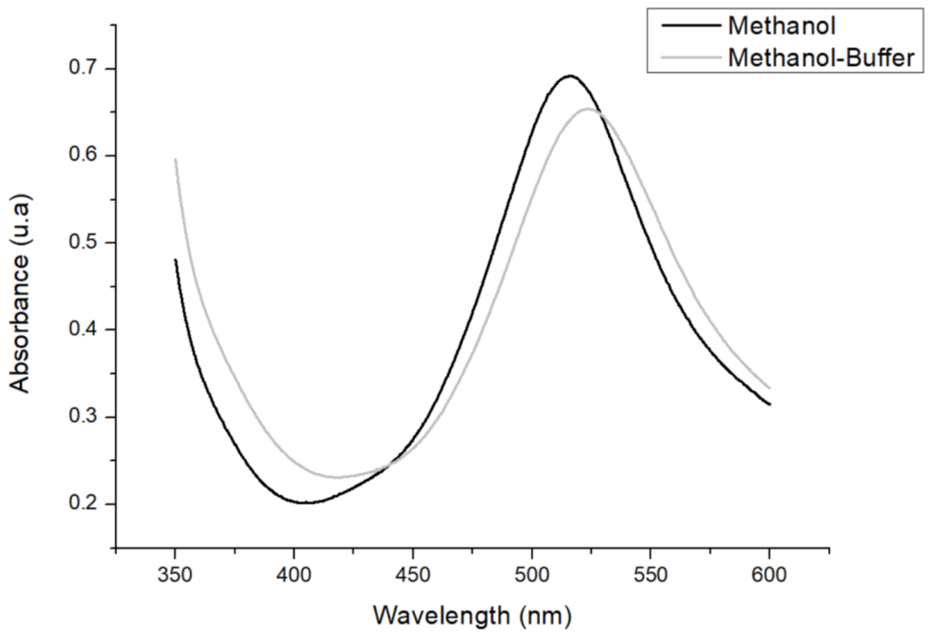

Figure 1. UV-Vis absorbance spectra of DPPH (25 mg/L) in methanol (**black**) and methanol-buffer (**grey**) (0.1 M of citric acid and 0.2 M of phosphate disodium, pH 3.6, final proportion 60% methanol and 40% buffer).

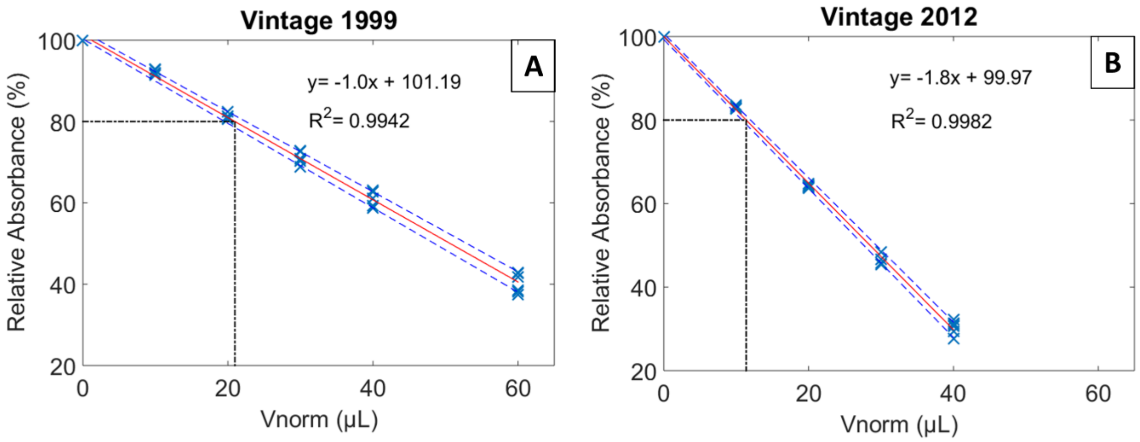

Figure 2. DPPH measurements of vintage 1999 (**A**) and 2012 (**B**) in methanol-buffer. The red line represents the regression line, and the two blue dashed lines the 95% confidence interval. The dot-dashed line guides the eye to EC_{20}, which is the volume of wine needed to decrease the initial absorbance of DPPH by 20%.
